# Angiotensin II type 2 receptor correlates with therapeutic effects of losartan in rats with adjuvant-induced arthritis

**DOI:** 10.1111/jcmm.12128

**Published:** 2013-09-23

**Authors:** Di Wang, Shanshan Hu, Jie Zhu, Jun Yuan, Jingjing Wu, Aiwu Zhou, Yujing Wu, Wendi Zhao, Qiong Huang, Yan Chang, Qingtong Wang, Wuyi Sun, Wei Wei

**Affiliations:** Institute of Clinical PharmacologyAnhui Medical University, Key Laboratory of Anti-inflammatory and Immune Medicine of China Education MinistryHefei, Anhui Province, China

**Keywords:** rheumatoid arthritis, adjuvant-induced arthritis, losartan, angiotensin II type 2 receptor

## Abstract

The angiotensin II type 1 receptor (AT1R) blocker losartan ameliorates rheumatoid arthritis (RA) in an experimental model. In RA, AT2R mainly opposes AT1R, but the mechanism by which this occurs still remains obscure. In the present study, we investigated the role of AT2R in the treatment of rats with adjuvant-induced arthritis (AIA) by losartan. Adjuvant-induced arthritis rats were treated with losartan (5, 10 and 15 mg/kg) and methotrexate (MTX; 0.5 mg/kg) *in vivo* from day 14 to day 28. Arthritis was evaluated by the arthritis index and histological examination. Angiotensin II, tumour necrosis factor-α, and VEGF levels were examined by ELISA. The expression of AT1R and AT2R was detected by western blot and immunohistochemistry analysis. After stimulation with interleukin-1β *in vitro*, the effects of the AT2R agonist CGP42112 (10^−8^–10^−5^ M) on the chemotaxis of monocytes induced by 10% foetal calf serum (FCS) were analysed by using Transwell assay. Subsequently, the therapeutic effects of CGP42112 (5, 10 and 20 μg/kg) were evaluated *in vivo* by intra-articular injection in AIA rats. After treatment with losartan, the down-regulation of AT1R expression and up-regulation of AT2R expression in the spleen and synovium of AIA rats correlated positively with reduction in the polyarthritis index. Treatment with CGP42112 inhibited the chemotaxis of AIA monocytes *in vitro*, possibly because of the up-regulation of AT2R expression. Intra-articular injection with CGP42112 (10 and 20 μg/kg) ameliorated the arthritis index and histological signs of arthritis. In summary, the present study strongly suggests that the up-regulation of AT2R might be an additional mechanism by which losartan exerts its therapeutic effects in AIA rats.

## Introduction

Rheumatoid arthritis is a chronic, systemic autoimmune disease that is characterized by the infiltration of various inflammatory cells and the uncontrolled synoviocytes hyperplasia in the synovium, ultimately leading to progressive and irreversible joint destruction. There are numerous therapeutic drug options for RA, such as non-steroidal anti-inflammatory drugs, disease-modifying anti-rheumatic drugs (DMARDs) and biological agents, including TNF inhibitors and anakinra. Although these drugs have shown efficacy in treating RA, they do not alter the fundamental mechanisms of RA disease progression 1. Furthermore, these drugs are often associated with inadequate responses and severe adverse reactions after a few years of use. Thus, these limitations call for the development of safer and more effective long-term therapeutic strategies for RA patients, in particular, interventions targeting angiotensin II and AT1R.

Angiotensin II is well known to act as a cardiovascular mediator by binding to AT1R in the circulation and has a primary role in the control of body fluid volume homoeostasis and blood pressure. It is now widely accepted that, as a pro-inflammatory mediator, angiotensin II contributes significantly to hypertension, renal disease and cardiovascular disease by exerting a pro-inflammatory effect *via* AT1R 2. However, reports in the last few years have described the presence and up-regulation of angiotensin-converting enzyme (ACE) in the peripheral blood and synovium samples obtained from patients with RA. This strongly suggests that a novel therapeutic strategy for RA could be achieved by limiting angiotensin II synthesis or blocking the interaction between angiotensin II and AT1R 3,4. Several studies have confirmed the clinical beneficial of ACE inhibitors (ACEIs) and/or AT1R blockers (ARBs) in RA experimental animal models, including AIA and collagen-induced arthritis 6–9.

Although AT1R is thought to be responsible for most of the physiological and pathological actions of angiotensin II, angiotensin II can also act through AT2R, which has counter-regulatory actions to AT1R. Intriguingly, in contrast to physiological conditions, AT2R expression is significantly increased in pathological circumstances, such as inflammation, ischaemia, trauma and infarction 10,11. In particular, when AT2R-knockout animals are exposed to disease models, loss of AT2R expression causes a deterioration of pathology compared with wild-type control animals 13. The results from *in vitro* and *in vivo* studies using AT2R agonists have revealed that the stimulation of AT2R inhibited proliferation, inflammation and remodelling, and induced vasodilation and regeneration. As such, it appeared to have a beneficial role in the disease condition 14–17. In contrast to AT1R, the role of AT2R in RA and AIA is less well understood.

Usually, AT1R blockade by ARBs, such as losartan, might lead to a feedback loop that increases free angiotensin II, consequently resulting in the stimulation of AT2R and resulting in anti-inflammatory and anti-fibrotic effects 18,19. Therefore, in the present study, we observed the change in AT2R expression *in vivo* in AIA rats treated with losartan. Additionally, we investigated the effects of direct AT2R stimulation on primary monocytes cultured from the peripheral blood of AIA rats *in vitro*. Finally, we evaluated the therapeutic effects of CGP42112, administered by intra-articular injection, on AIA rats *in vivo*.

## Materials and methods

### Reagents

The losartan potassium was purchased from Hangzhou Merck Pharmaceutical Co., Ltd (Hangzhou, China), and the peptide AT2R agonist, CGP42112, was purchased from Tocris Bioscience (Bristol, UK). Methotrexate was purchased from Shanghai Xinyi Pharmaceutical Co., Ltd (Shanghai, China). Triamcinolone acetonide (TA) was purchased from Shanghai General Pharmaceutical Co., Ltd (Shanghai, China). rhIL-1β was obtained from Peprotech (Rocky Hill, NJ, USA). The mouse monoclonal primary antibody to AT1R protein and the rabbit polyclonal primary antibody to AT2R protein were from Abcam (Hong Kong, China). HyClone FCS and Gibco DMEM were obtained commercially from Thermo Scientific (Rockford, MN, USA) and Life Technologies (New York, NY, USA), respectively.

### Animals

Male Sprague-Dawley rats weighing 180 ± 20 g were purchased from the Experimental Animal Center of Anhui Medical University (GradeII, Certificate No. 2012-0012). The animals were housed in a specific pathogen-free room with controlled ambient temperature (22 ± 2°C) and humidity (50 ± 10%), with food and water *ad libitum*. The animals were acclimated to the housing conditions for 3 days prior to the experiments. All experimental protocols described in the present study were approved by the Ethics Review Committee for Animal Experiment, Anhui Medical University.

### AIA induction

Complete Freund’s adjuvant (CFA) was prepared by suspending heat-killed *Mycobacterium butyricum* (National Vaccine and Serum Institute, Beijing, China) in liquid paraffin at 10 mg/ml. As described previously 21–22, the AIA model was generated by immunizing the rats with a single intradermal injection of 0.1 ml CFA into the right hind metatarsal rat footpad. The day of CFA injection was designated day 0, and the secondary polyarthritis reaction occurred on ∼day 13. Sham-operated rats were injected with 0.1 ml normal saline (NS).

### Treatment

Adjuvant-induced arthritis rats were randomly assigned to the following treatments: losartan (5, 10 and 15 mg/kg/day, orally, once daily), MTX (0.5 mg/kg, intraperitoneally, per 3 days), and vehicle (NS). Sham-operated rats received the vehicle (NS). The treatment started on day 14 after immunization and continued until day 28. After 15 days of treatment, on day 28, all rats were anesthetized with the chloral hydrate and killed (*n* = 6 per group). The serum, paw and spleen were collected for further study. To study the effects of AT2R agonist on rats with AIA, CGP42112 (5, 10 and 20 μg/kg, once every 3 days, total three times) and TA (1 mg/kg, once every 3 days, total three times) were administered by intra-articular injection into the left hind (non-injected) paws following the onset of the secondary arthritis (*n* = 7 per group). Normal and model rats received NS instead.

### Arthritis assessment

The arthritic severity in each paw was evaluated by using a macroscopic scoring system ranging from 0 to 4 as follows: 0 = paws with no swelling and focal redness; 1 = paws with swelling of finger joints; 2 = paws with mild swelling of ankle or wrist joints; 3 = paws with severe inflammation of the entire paws; and 4 = paws with deformity or ankylosis. The cumulative score for all four paws of each rat was used as the polyarthritis index, with a maximum value of 16. The rats were examined on days 14, 18, 22 and 26 by two independent observers with no knowledge of the treatment protocol. The arthritis index, with a maximum value of four, was used to evaluate the effects of intra-articular injection of CGP42112 on secondary arthritis in the single left hind (non-injected) paw of AIA rats.

### Histological examination

The left (non-injected) hind paws were amputated above the ankle joints and were fixed in 10% neutral-buffered formalin, then decalcified in 5% formic acid and embedded in paraffin. The sections were stained with haematoxylin and eosin and were examined microscopically. Synovium proliferation was graded as follows: Grade 0, proliferation was absent; Grade 1, proliferation was mild with two to four layers of reactive synoviocytes; Grade 2, proliferation was moderate with four plus layers of reactive synoviocytes, increased mitotic activity and mild or absent synovial cell invasion of the adjacent bone and connective tissue; and Grade 3, proliferation was severe and characterized by invasion and effacement of the joint space and adjacent cartilage, bone, and connective tissue. Cellular infiltration was graded as follows: Grade 0, no changes; Grade 1, few focal infiltrates; Grade 2, extensive focal infiltrates; and Grade 3, extensive infiltrates invading the capsule with aggregate formation. Cartilage erosion was graded as follows: Grade 0, no changes; Grade 1, superficial, localized cartilage degradation in more than one region; Grade 2, localized deep cartilage degradation; and Grade 3, extensive deep cartilage degradation at several locations. Pannus formation was graded as follows: Grade 0, no changes; Grade 1, pannus formation at up to two sites; Grade 2, pannus formation at up to four sites, with infiltration or flat overgrowth of the joint surface; and Grade 3, pannus formation at more than four sites or extensive pannus formation at two sites.

### ELISA

The synovium homogenate was prepared, and after centrifugation for 10 min. at 24°C and 560 × g, the supernatant of the homogenate and serum were collected for ELISA analysis. The production of TNF-α, VEGF, and angiotensin II in the serum and synovium was determined by ELISA, according to manufacturer’s instructions. The absorbance at 450 nm was measured colorimetrically on a microplate reader (BioTek Elx×808, Winooski, VT, USA), according to the manufacturer’s protocol. The concentration was calculated from the absorbance reading at 450 nm. The angiotensin II, TNF-α, and VEGF ELISA kits were purchased from CUSABIO (Wuhan, China) and R&D Systems (Palo Alto, CA, USA), respectively.

### Western blot analysis

Splenocytes were obtained from the spleen by gentle sieving through a sterile nylon screen. They were then placed in haemolysis buffer at 24°C for 5 min. For western blot analysis, splenocytes, as well as monocytes isolated from rat peripheral blood, were washed twice with ice-cold PBS, and subsequently lysed in RIPA lysis buffer for 30 min. on ice. The synovium homogenate was prepared on ice by using the RIPA lysis buffer. After centrifugation for 20 min. at 4°C and 5000 × g, the supernatant of the homogenate was collected for western blot analysis. The concentration of the extracted protein was determined by using the Lowry assay. A mixture of the lysates with the loading buffer (1:4) was heated for 10 min. at 95°C. The protein samples were resolved by SDS-PAGE, transferred to polyvinylidene fluoride membranes (Millipore, Billerica, MA, USA) and incubated with blocking buffer for 2 hrs at 37°C. Immunoblotting was performed with the AT1R and AT2R primary antibodies at 1/100 dilution. The membranes and antibodies were incubated overnight at 4°C, followed by incubation with the appropriate Horseradish peroxidase-conjugated secondary antibody. Immunodetection was carried out with enhanced chemiluminescence (Pierce, Rockford, IL, USA) and the Imagequant LAS4000 mini system (GE, Pittsburgh, PA, USA). The western blot results were quantitatively analysed by optical density by using the Image J software.

### Isolation and culture of rat peripheral blood monocytes

Heparinized peripheral blood was collected from anesthetized rats. As described previously 23, the blood was layered on a Ficoll-Hypaque density gradient (Solarbio, Beijing, China), then centrifuged at 563 × *g* for 20 min. at 24°C. The interface between the plasma and Ficoll was then collected, and washed three times with PBS to remove the platelets. After centrifugation for 10 min. at 24°C and 317 × g, the monocyte and lymphocyte fractions were collected, washed in PBS and cultured in standard culture medium for 2 hrs. As the monocytes are known to be adherent, the culture plate was then washed with DMEM to remove the lymphocytes.

The monocytes were isolated with 0.02% EDTA/PBS and were 90% pure, as evaluated by anti-CD14-PE staining (BD Biosciences Pharmingen, San Diego, CA, USA) and flow cytometry (Beckman Coulter, Indianapolis, IN, USA). The monocytes were suspended in DMEM containing 10% FCS, 50 μg/ml streptomycin and 50 units/ml penicillin at 37°C in 5% CO_2_/95% air in a humidified atmosphere. In the *in vitro* study, primary monocytes obtained from AIA rats were pre-stimulated with interleukin (IL) -1β at a final concentration of 10 μg/l for 2 hrs. They were then incubated with or without losartan (10^−6^ M) or the AT2R agonist, CGP42112.

### Immunohistochemistry

Monocytes (concentration of 5 × 10^7^cells/l) were seeded onto glass cover slips in 12-well plates and allowed to adhere overnight. The following day, the cover slip was washed with PBS and fixed in 4% paraformaldehyde solution for 30 min. at 24°C. The monocytes were then blocked with goat serum for 20 min. prior to incubation with the AT1R and AT2R primary antibodies at 1/100 dilution. The samples were incubated overnight at 4°C and PBS was used as a negative control. Immunohistochemistry (IHC) staining was performed according to the protocol in the SP9000 IHC reagents kit (ZSGB Bio, Beijing, China). For each sample, six fields were randomly observed by microscopy (Olympus BX53 system, Center Valley, PA, USA) (×1000). The IHC results were quantitatively analysed by optical density by using the Image-ProPlus Software.

### Survival assay

A Cell Counting Kit-8 (WST-8) (Dojindo Laboratories, Kumamoto, Japan) was used to explore whether AT2R stimulation by CGP42112 inhibited the survival of monocytes. Monocytes were added to each well of a 96-well culture plate at a cell concentration of 10^8^ cells/l in 100 μl DMEM containing 10% FCS and allowed to adhere overnight. The following day, the cells were exposed to various concentrations of CGP42112, ranging from 10^−10^ to 10^−5^ M. After 5 days, 10 μl WST-8 solution was added to each well and the samples were incubated for 3 hrs 24. After 3 hrs, the absorbance at 450 nm was measured colorimetrically on a microplate reader (BioTek Elx×808), according to the manufacturer’s protocol.

### Chemotaxis assay

The chemotaxis of the monocytes was assayed by using Transwell chambers (Corning, NY, US) with a filter of 6.5 mm diameter and 8.0 μm pore size. DMEM containing 10% FCS as a chemoattractant factor was placed in the lower wells, and monocytes were suspended at a concentration of 5 × 10^7^cells/l in serum-free DMEM in the upper wells. As described previously 25, the chamber was incubated at 37°C for 2 hrs to allow the cells to attach, after which the monocytes were treated with the indicated concentrations of CGP42112 and losartan. After incubation for 4 hrs, the non-migrating cells were removed from the upper surface of the filter by using a cotton swab. The filters were fixed in methanol for 15 min. and stained with 0.1% crystal violet for 1 min. Chemotaxis was quantified by counting the stained cells that had migrated to the lower side of the filter by using a microscope (Olympus BX53 system) (×400). For each assay, six random fields were assessed.

### Statistical analysis

Statistical analysis was performed by Student’s *t*-test or anova by using SPSS 11.5 software (Chicago, IL, USA). anova was used exclusively for multi-group comparisons, and Student’s *t*-test was only used for independent, two-group comparisons. The correlation coefficient was used to examine the correlation between the polyarthritis index and the expression of AT1R and AT2R. The data are presented as the means ± SD and the differences were considered statistically significant at *P* < 0.05.

## Results

### Losartan ameliorated the arthritis assessment and histological manifestation

**Figure 1:**
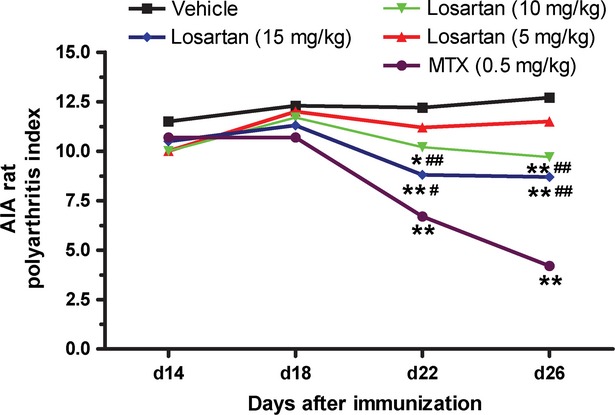
Losartan ameliorated the polyarthritis index in adjuvant-induced arthritis (AIA) rats. The secondary polyarthritis reaction was induced on day 13, and the polyarthritis index was evaluated on days 14, 18, 22 and 26. Treatment with losartan (10 and 15 mg/kg) significantly decreased the polyarthritis index on days 22 and 26. *P < 0.05, **P < 0.01 *versus* AIA rats. ^#^*P* < 0.05, ^##^*P* < 0.01 *versus* methotrexate+AIA rats. The data are presented as the means ± SD (*n* = 6).

After the secondary polyarthritis reaction was successfully induced on day 13, the polyarthritis index was evaluated on days 14, 18, 22 and 26. The results are summarized in Figure 1. In agreement with previous published reports, treatment of AIA rats with either MTX or losartan (10 and 15 mg/kg) significantly decreased the polyarthritis index on days 22 and 26 compared with treatment with vehicle. In addition, losartan-treated AIA rats exhibited a less significant reduction in the polyarthritis index, compared with MTX-treated AIA rats.

**Figure 2:**
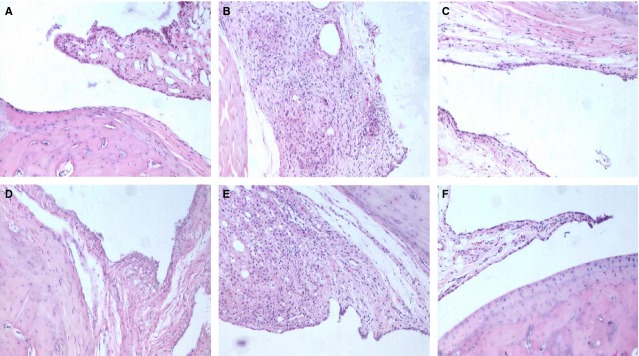
Losartan ameliorated the histological manifestation of arthritis in adjuvant-induced arthritis (AIA) rats. Normal (A). The joint histopathology of AIA rats was characterized by marked synoviocyte proliferation and dense mononuclear cells infiltration (B). The administration of losartan (15 and 10 mg/kg) (C and D, respectively) or methotrexate (F) significantly alleviated these abnormalities in the joints of AIA rats. The changes were not significant with treatment with losartan (5 mg/kg) (E).

The left hind paw was examined histologically for the intensity hyperplasia of the lining layer, mononuclear cell infiltration, pannus formation and cartilage erosion. The characteristic histopathology of arthritis was observed in AIA rats, specifically, marked proliferation of synoviocytes and infiltration of dense mononuclear cells. These abnormalities were alleviated significantly after the administration of MTX or losartan (10 and 15 mg/kg) in AIA rats (Fig. 2).

### Losartan decreased TNF-α, VEGF and angiotensin II levels

**Figure 3:**
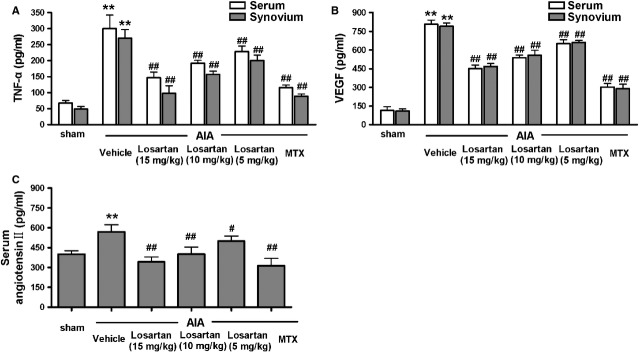
Losartan decreased the production of tumour necrosis factor-α (TNF-α), VEGF and angiotensin II. Treatment with losartan significantly decreased TNF-α (A) and VEGF (B) in the serum and synovium of adjuvant-induced arthritis (AIA) rats. Losartan also suppressed the serum angiotensin II level (C) in AIA rats. **P < 0.01 *versus* sham-operated rats. ^#^*P* < 0.05, ^##^*P* < 0.01 *versus* AIA rats. The data are presented as the means ± SD (*n* = 6).

The pro-inflammatory mediators, elevated TNF-α and VEGF contribute largely to the pathogenesis of RA and in experimental arthritis models. Similarly, treatment with either MTX or losartan (5, 10 and 15 mg/kg) significantly reduced TNF-α and VEGF levels in the serum and synovium of AIA rats (Fig. 3A and B). In contrast to classical pro-inflammatory mediators, such as TNF-α and VEGF, the role of angiotensin II remains controversial because the effect evoked by angiotensin II depends on the AT1R/AT2R ratio. In the present study, the levels of angiotensin II in the serum of AIA rats increased significantly compared with that in sham-operated rats, but the treatment with MTX or losartan (5, 10 and 15 mg/kg) still caused a significant suppression in the level of angiotensin II in the serum of AIA rats (Fig. 3C).

### Losartan-induced effects on AT1R and AT2R expression

**Figure 4:**
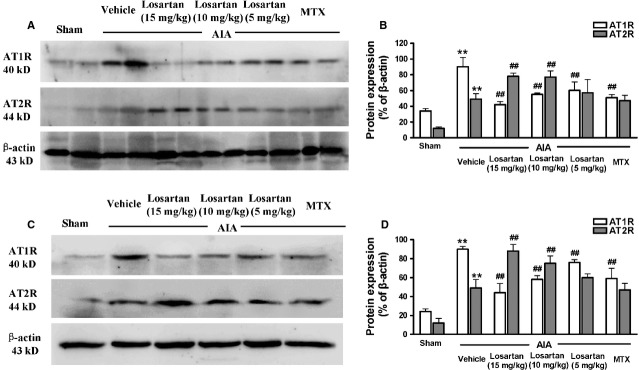
Losartan decreased angiotensin II type 1 receptor (AT1R) expression and up-regulated AT2R expression. AT1R and AT2R expressions were both significantly increased in the splenocytes (A and B) and synovium (C and D) of adjuvant-induced arthritis (AIA) rats. The western blot result showed down-regulation of AT1R expression and up-regulation of AT2R expression within the splenocytes (A and B) and synovium (C and D) of AIA rats after treatment with losartan. **P < 0.01 *versus* sham-operated rats. ^##^*P* < 0.01 *versus* AIA rats. The data are presented as the means ± SD (*n* = 6).

First, we explored AT1R expression in freshly isolated splenocytes and synovium. The level of AT1R was significantly increased in the vehicle group compared with sham-operated group. As an AT1R blocker agent, losartan (5, 10 and 15 mg/kg) significantly suppressed AT1R expression in the splenocytes and synovium of AIA rats. We also observed that the AT2R expression was also significantly increased in the vehicle group compared with sham-operated group. Conversely, treatment with losartan (5, 10 and 15 mg/kg) significantly increased the expression of AT2R in the splenocytes and synovium of AIA rats (Fig. 4). Interestingly, as a representative DMARDs agent, MTX had a similar effect on AT1R level as losartan, but had no significant influence on AT2R expression. We found that the down-regulation of AT1R expression and the up-regulation of AT2R expression in the splenocytes and synovium of AIA rats correlated positively with a reduction in the polyarthritis index, as determined by correlation analysis (Table 1).

**Table 1:** The correlation between the polyarthritis index and expression of AT1R and AT2R

	Regression equation	Correlation coefficient	*P*-value
AT1R			
Splenocytes	*y* = 7.2669*x* + 5.7937	0.7821	0.0010
Synovium	*y* = 7.104*x* + 6.1702	0.7835	0.0026
AT2R			
Splenocytes	*y* = −13.042*x* + 18.697	0.8709	0.0002
Synovium	*y* = −10.396*x* + 17.416	0.7718	0.0014

The correlation analysis showed that the down-regulation of AT1R (angiotensin II type 1 receptor) expression and the up-regulation of AT2R (angiotensin II type 2 receptor) expression in the spleen and synovium of adjuvant-induced arthritis rats correlated positively with a reduction in the polyarthritis index.

### AT1R and AT2R expression in monocytes cultured *in vitro*

**Figure 5:**
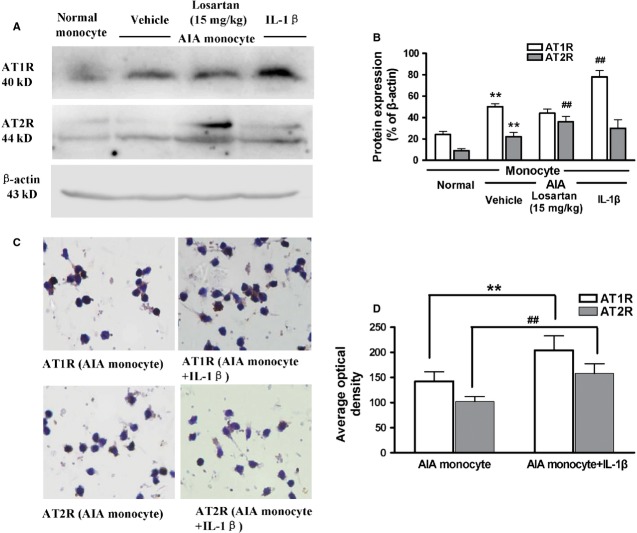
Angiotensin II type 1 receptor (AT1R) and AT2R expression in monocytes cultured *in vitro*. The western blot result showed the expressions of AT1R and AT2R in monocytes cultured *in vitro*, including monocytes were obtained from normal rats, adjuvant-induced arthritis (AIA) rats, losartan (15 mg/kg)-treated AIA rats and interleukin (IL)-1β-stimulated AIA monocytes (A and B). **P < 0.01 *versus* sham-operated rats. ^##^*P* < 0.01 *versus* AIA rats. The data are presented as the means ± SD (*n* = 6). By immunohistochemistry, AT1R and AT2R expressions were observed to be significantly up-regulated in IL-1β-stimulated AIA monocytes (C and D). **P < 0.01. ^##^*P* < 0.01. The data are presented as the means ± SD (*n* = 6).

We assessed the expression of AT1R and AT2R in monocytes cultured *in vitro*, specifically, monocytes isolated from normal rats, AIA rats, losartan (15 mg/kg)-treated AIA rats and IL-1β-stimulated AIA monocytes, respectively (Fig. 5A and B). The western blot result showed that the expression of AT1R and AT2R both increased significantly in AIA monocytes compared with normal monocytes. AT2R expression in the monocytes from losartan-treated AIA rats was significantly higher compared with AIA monocytes, and AT1R expression in IL-1β-stimulated AIA monocytes was significantly higher compared with AIA monocytes. Additionally, as shown in Figure 5C and D, by IHC, we determined that the levels of AT1R and AT2R were significantly up-regulated in IL-1β-stimulated AIA monocytes.

### Treatment with CGP42112 inhibited monocytes chemotaxis

**Figure 6:**
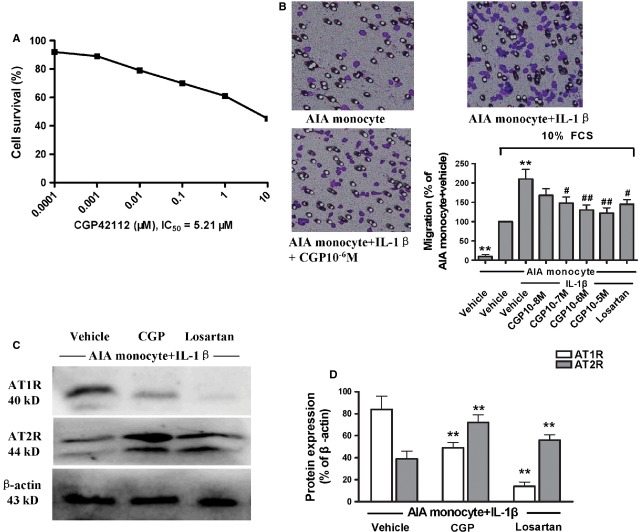
The effects of CGP42112 on monocytes cultured *in vitro*. The IC_50_ value of CGP42112 was 5.21 × 10^−6^ M (A). Treatment with CGP42112 (10^−7^–10^−5^ M) suppressed significantly the chemotaxis of interleukin (IL) -1β-stimulated adjuvant-induced arthritis (AIA) monocytes (B). **P < 0.01 *versus* AIA monocytes. ^#^*P* < 0.05, ^##^*P* < 0.01 *versus* IL-1β-stimulated AIA monocytes. The data are presented as the means ± SD (*n* = 6). Treatment with CGP42112 (10^−6^ M) or losartan (10^−6^ M) significantly increased angiotensin II type 2 receptor (AT2R) expression, and also decreased AT1R expression (C and D). **P < 0.01 *versus* IL-1β-stimulated AIA monocytes. The data are presented as the means ± SD (*n* = 6).

As shown by the inhibitory curve corresponding to various concentrations of CGP42112 (Fig. 6A), the inhibitory rates of CGP42112 were all above 50%, except 10^−5^ M CGP42112. As determined by using SPSS 11.5 software, the IC_50_ value of CGP42112 on IL-1β-stimulated AIA monocytes was 5.21 × 10^−6^ M. To explore the inhibitory effect of AT2 stimulation by CGP42112 on chemotaxis in IL-1β-stimulated AIA monocytes, we chose to focus on concentrations of CGP42112 ranging from 10^−18^ to 10^−5^ M. In the *in vitro* study, co-incubation with 10% FCS and/or IL-1β caused a significant increase in the chemotaxis of AIA monocytes. This was significantly suppressed by treatment with CGP42112 (10^−7^–10^−5^ M) or losartan (10^−6^ M; Fig. 6B).

### Effects of CGP42112 on monocytes AT1R and AT2R expression

As shown in Figure 6C and D, the western blot results indicated that direct AT2R stimulation by CGP42112 (10^−6^ M) significantly increased AT2R expression in IL-1β-stimulated AIA monocytes compared with vehicle-treated cells. Losartan (10^−6^ M) also caused a significant elevation in AT2R expression. In contrast, treatment with CGP42112 (10^−6^ M) or losartan (10^−6^ M) both caused a significant reduction in AT1R expression in IL-1β-stimulated AIA monocytes.

### Therapeutic effects of intra-articular injection of CGP42112 in AIA rats

**Figure 7:**
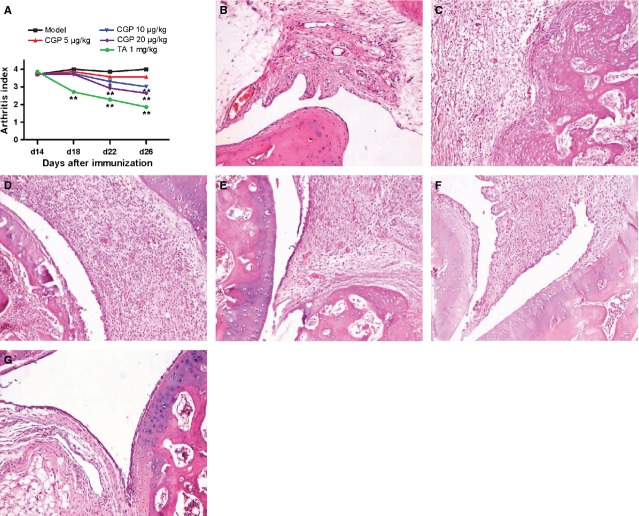
The therapeutic effects of CGP42112 on adjuvant-induced arthritis (AIA) rats *in vivo*. Intra-articular injection of CGP42112 (10 and 20 μg/kg) significantly decreased the arthritis index of the left hind paw on days 22 and 26 (A). *P < 0.05, **P < 0.01 *versus* model group. The data are presented as the means ± SD (*n* = 7). The histological appearance of ankle joint from normal rat (B). The AIA rats had secondary synovitis, which was characterized by marked synoviocytes proliferation, dense mononuclear cells infiltration, pannus formation and cartilage erosion (C). Intra-articular injection with CGP42112 (5 μg/kg) did not ameliorate secondary synovitis (D). In contrast, the histological abnormalities in the ankle joints from CGP42112 (10 and 20 μg/kg)-treated rats and TA (1 mg/kg)-treated rats were significantly alleviated (E–G, respectively).

As summarized in Figure 7A, intra-articular injection of AIA rats with CGP42112 (10 and 20 μg/kg) significantly decreased the arthritis index of single left hind (non-injected) paw on days 22 and 26, and treatment with TA (1 mg/kg) significantly decreased the arthritis index on days 18, 22 and 26. Histological examination revealed that treatment with CGP42112 (10 and 20 μg/kg) and TA (1 mg/kg) ameliorated secondary synovitis in the AIA rats left hind (non-injected) paw (Fig. 7B–G and Table 2).

**Table 2:** Histological evaluation of ankle joints from CGP42112-treated AIA rats

	Synovium proliferation	Cellular infiltration	Cartilage erosion	Pannus formation
Model	2.714 ± 0.487	2.857 ± 0.377	2.571 ± 0.534	2.428 ± 0.534
CGP42112 (5 μg/kg)	2.571 ± 0.534	2.714 ± 0.487	2.714 ± 0.487	2.428 ± 0.534
CGP42112 (10 μg/kg)	2.142 ± 0.377*	2.142 ± 0.377*	2.285 ± 0.487	2.142 ± 0.377
CGP42112 (20 μg/kg)	1.714 ± 0.487**	1.714 ± 0.755**	1.571 ± 0.534**	1.428 ± 0.534**
TA (1 mg/kg)	1 ± 0.577**	1.285 ± 0.487**	0.857 ± 0.377**	1 ± 0.577**

* *P* < 0.05

** *P* < 0.01 *versus* model group.

Histological appearance was scored by the presence of synovium proliferation, cellular infiltration, pannus formation and cartilage erosion.

The data are presented as the means ± SD (*n* = 7).

AIA: adjuvant-induced arthritis; TA: triamcinolone acetonide.

## Discussion

Martin *et al*. reported in the early 1980s that the ACEI captopril was clinically beneficial in the treatment of RA 26. However, the clinical benefit of this drug was subsequently attributed to the presence of a thiol group in the compound structure, which was similar to penicillamine, and not to ACE inhibition 27. More recently, a series of reports have demonstrated that the pro-inflammatory properties of angiotensin II are mediated by AT1R and that non-thiol ACEIs and AT1R blockade can have therapeutic benefits in the treatment of RA and other autoimmune diseases 6–30. Furthermore, ACEI and ARB have been widely used for treating hypertension in many countries, and as such, these drugs possess additional benefits of reducing high cardiovascular risks in RA patients 31–32. Additionally, Wruck *et al*. reported that oxidative stress is significantly involved in cartilage degradation in experimental arthritis models 33 and that the beneficial effects of losartan in renovascular hypertension are partly because of the preferential reduction in oxidative stress 34. Pufe *et al*. reported that the expression of VEGF protein and receptor is strongly increased in rheumatoid synovium 35 and that the combined therapy of MTX and losartan decreased the level of VEGF in the serum of AIA rats 6. Given these data, a randomized, placebo-controlled trial of ARB is warranted to fully evaluate the importance of the angiotensin pathway in RA 7.

It is well established that AT1R accounts for the majority of effects evoked by angiotensin II, including vasoconstriction, pro-inflammation, pro-fibrosis and growth promotion. In addition to AT1R, angiotensin II has another receptor subtype, AT2R, which mainly opposes the action of AT1R. This suggests that the stimulation of AT2R might have therapeutic potential. It has been reported that counter-activation of AT2R in response to AT1R blockade contributes to anti-inflammatory effects, suggesting that the losartan-induced treatment effects in RA and experimental arthritis models may be attributed to AT2R. In the present study, losartan ameliorated the symptoms of arthritis in AIA rats. Additionally, we found that treatment with losartan not only suppressed AT1R expression but also increased AT2R expression in the splenocytes and synovium. Furthermore, the down-regulation of AT1R expression and up-regulation of AT2R expression within the splenocytes and synovium of AIA rats correlated positively with a reduction in the polyarthritis index. ELISA results showed that the levels of angiotensin II in the serum of losartan (5, 10 and 15 mg/kg)-treated AIA rats were significantly lower than those in vehicle-treated AIA rats. This may be due to the diversion of angiotensin II from AT1R, which is blocked, to AT2R, which is available, thus resulting in a reduction in angiotensin II levels. However, the effects of direct AT2R stimulation in AIA rats are not known.

Monocytes, macrophages and dendritic cells produce angiotensin II *via* ACE and express AT1R and AT2R 36. It has been reported that ACE activity is up-regulated in unstimulated peripheral blood monocytes from most RA patients 3. In the present study, the presence and up-regulation of AT1R and AT2R were assessed in normal monocytes, AIA monocytes and IL-1β-stimulated AIA monocytes. Does direct AT2R stimulation inhibit IL-1β-stimulated AIA monocytes *in vitro*? In the present study, we observed that treatment with the AT2R agonist, CGP42112, effectively inhibited the chemotaxis of IL-1β-stimulated AIA monocytes *in vitro*. Western blot analysis showed that the administration of CGP42112 led to the up-regulation of AT2R and down-regulation of AT1R within stimulated AIA monocytes. Similarly, losartan also exerted an inhibitory effect on chemotaxis in IL-1β-stimulated AIA monocytes. However, treatment with losartan up-regulated AT2R expression and decreased AT1R expression.

Even though elevated AT2R plays a beneficial role in arthritis progression, the protective actions of AT2R does not prevent AIA disease *in vivo*. A possible reason is that even though both AT1R and AT2R expressions were significantly up-regulated under AIA, as compared with that in sham-operated rats, the expression of AT2R was still significantly lower than that of AT1R. Because AT1R and AT2R have a similar affinity for angiotensin II, we speculate that the significantly higher level of AT1R may account for the predominance of its pathological role in arthritis, totally offsetting the AT2R-mediated beneficial effects evoked by angiotensin II. To further confirm the direct effects of AT2R stimulation on AIA rats *in vivo*, the therapeutic effects of intra-articular injection with CGP42112 were evaluated. We found that treatment with CGP42112 effectively ameliorated the arthritis index and histological signs of arthritis.

In summary, the present study showed that the losartan-induced therapeutic effects on AIA rats *in vivo* might be correlated with the up-regulation of AT2R and the down-regulation of AT1R. Direct stimulation of AT2R by CGP42112 exerted an inhibitory effect on IL-1β-stimulated AIA monocytes *in vitro* and provided a therapeutic effect on AIA rats *in vivo*. The clinical application of AT2R stimulation in RA therapy still requires further translational and clinical studies before it can be implemented as the peptide AT2R agonist CGP42112 is not suitable for *in vivo* oral use, as it is rapidly degraded *in vivo*, and it has both agonistic and antagonistic properties. This present study strongly suggests that the up-regulation of AT2R might be an additional mechanism by which losartan exerts its therapeutic effects in AIA rats.
